# A case series and literature review of infections due to *Myroides* spp.: identification of contributing factors and emerging antibiotic susceptibility trends

**DOI:** 10.1099/acmi.0.000549.v2

**Published:** 2023-05-05

**Authors:** Uzair Khan, Ellora Pandey, Nageswari Gandham, Nikunja Das, Sahjid Mukhida, Sriram Kannuri, Shalini Bhaumik, Shahzad Mirza

**Affiliations:** ^1^​ Department of Microbiology, Dr. D. Y. Patil Medical College, Hospital and Research Centre, Dr. D.Y. Patil Vidyapeeth, Pune, India

**Keywords:** *Flavobacteriaceae *infections, *Myroides*, multiple drug resistance, catheter-related infections, case report

## Abstract

**Introduction.:**

Infections forby *Myroides* spp. can lead to significant morbidity and mortality, particularly in immunocompromised patients with underlying co-morbidities. Recent reports have highlighted its intrinsic and acquired drug resistance, making it a particularly challenging infectious agent to combat.

**Methods.:**

*
Myroides* spp. isolated and reported in clinically significant urine samples were considered for the study. Identification of the organism was done via the VITEK 2C system. Antibiotic susceptibility testing was done using both manual and automated methods following Clinical and Laboratory Standards Institute (CLSI) guidelines. Existing literature was searched on MEDLINE using PubMed.

**Results.:**

We present a series of five catheter-associated urinary tract infections due to *Myroides odoratimimus *, with sensitivity to only minocycline. This is the first case from Western India, and the third case in the existing literature that shows *Myroides* sensitivity only to minocycline. Our literature review is the first to systematically describe contributory factors to infection, allowing us to devise a clinically relevant tool that delineates contributory factors and efficacious drugs in *Myroides* spp*.* infection.

**Conclusion.:**

*
Myroides* spp*.* infections, previously considered rare and opportunistic, need cognizance and diagnostic suspicion especially in particular associated conditions.

## Data Summary

Anonymized individual participant data and other study documents can be requested for further research by contacting the corresponding author.

## Introduction

The genus *Myroides* belongs to the family *Flavobacteriaceae* and is present ubiquitously in the environment [1]. Clinically relevant species include *Myroides odoratus* [2–15], *M. odoratimimus* [16–30], *M. injenensis* [31, 32] and *M. phaeus* [33]. Previously considered ‘low-grade’ and not highly pathogenic, infections by *Myroides* spp. can lead to significant morbidity and mortality, particularly in immunocompromised patients with underlying co-morbidities [32]. These opportunistic pathogens have been implicated in both hospital- and community-acquired infections and affect a variety of sites (Table 1) [34]. Clinicians need to be aware of this pathogen, which is now being detected at a greater frequency possibly due to widespread availability of automated technologies. Recent reports have highlighted its intrinsic and acquired drug resistance, making it a particularly challenging infectious agent to combat [15, 25, 28, 30, 35–37]. Of particular interest is in the intensive care setting, where the likelihood of both infection and drug resistance is higher. Here we present a case series of five patients with extensively drug-resistant *M. odoratimimus* infection encountered at a tertiary care centre. We also review available literature on this subject, and this paper provides the first systematic description of possible contributory factors to infection along with trends in antibiotic susceptibility.

**Table 1.** Review of existing literature

Reference/year	* Myroides* species	No. of cases	Clinical infection	Possible contributory factors	Immune status*	Sensitive drugs on AST
Holmes/1979	*odoratum*	5	1. Stump infection, 2. Indwelling catheter infection, 3. Foot gangrene, cellulitis, 4 and 5. Recurrent UTI	1. Ischaemic lower limb disease, 2. Indwelling catheter, 3. Frostbite, 4. Bladder carcinoma, 5. CKD	na	S: sulfamethoxazole, cotrimoxazole, cephaloridine and nalidixic acid
Davis/1979	*odoratum*	1	Foot gangrene	Chronic alcoholic, possibly cirrhotic	na	S: chloramphenicol
MacFarlane/1985	*odoratum*	1	Ventriculitis	Prolonged hospitalization, multiple invasive procedures	na	S: cefotaxime
Hsueh/1995	*odoratum*	1	Bacteraemia, necrotizing fasciitis	Chronic HBV cirrhosis	−	S: aztreonam, imipenem, chloramphenicol, vancomycin, ofloxacin, ciprofloxacin
Ferrer/1995	*odoratum*	1	Bacterial endocarditis	CKD on chronic haemodialysis, invasive procedure	−	S: netilmicin, ceftazidime
Bachman/1996	*odoratum*	1	Cellulitis, bacteraemia	Chronic steroid use, unsanitary water exposure	−	S: ceftriaxone, cotrimoxazole, piperacillin, imipenem
Spanik/1998	*odoratum*	4	Bacteraemia	3 blood cancers with neutropenia, 1 solid cancer; 4 indwelling central venous catheter	3−, 1+	S: gentamicin, amikacin, ofloxacin, ciprofloxacin, netilmicin, tobramycin, azlocillin; only one patient was resistant to third-generation cephalosporins
Yağci/2000	*odoratimimus*	13	Pyuria	4 urinary tract neoplasia, 9 urinary calculi, 13 indwelling urinary catheter	+	R: amikacin, aztreonam, cefoperazone, ceftazidime, ceftriaxone, ciprofloxacin, gentamicin, imipenem, piperacillin, tetracycline, tobramycin and cotrimoxazole
Green/2001	*odoratus*	1	Recurrent cellulitis, bacteraemia,	Unsanitary water exposure	+	S: cotrimoxazole, piperacillin/tazobactam, ciprofloxacin and levofloxacin
Motwani/2004	*odoratum*	1	Cellulitis, septic shock	Diabetes mellitus, peripheral vascular disease	+	S: cotrimoxazole and fluoroquinolones
Thomas/2007	* Myroides* spp.	1	Acalculous cholecystitis	−	+	na
Bachmeyer/2008	*odoratimimus*	1	Cellulitis	Trauma; chronic alcoholic, possibly cirrhotic	−	S: ciprofloxacin, rifampicin
Benedetti/2011	*odoratimimus*	1	Septic shock, pneumonia, soft tissue infection	Trauma, environmental exposure, prolonged hospitalization, multiple invasive procedures	+	S: imipenem, meropenem, piperacillin/tazobactam, and ticarcillin/clavulanate
Ktari/2012	*odoratimimus*	7	4 UTI, 3 bladder colonization	4 indwelling D-J stent; 7 prolonged hospitalization, postoperative	+	Pan-drug resistant
Maraki/2012	*odoratimimus*	1	Cellulitis	Animal bite	+	S: levofloxacin, gatifloxacin, moxifloxacin, ofloxacin, cotrimoxazole, chloramphenicol, and amoxicillin/clavulanic acid
Kim/2012	*injenensis*	1	UTI	Multiple surgical procedures (radical hysterectomy and percutaneous nephrostomy), cervical cancer, CKD	+	na
Deepa/2014	*odoratus*	1	Pneumonia (secondary)	Diabetic, pulmonary tuberculosis	−	S: ciprofloxacin, levofloxacin, cotrimoxazole, amikacin, tobramycin, imipenem, meropenem and piperacillin/tazobactam
Crum-cianflone/2014	*odoratu*s	1	Necrotizing fascitis	Chronic HCV cirrhosis	−	S: meropenem
Endicott-Yazdani/2015	*odoratimimus*	1	Bacteraemia	Exposure to unsanitary water, trauma, diabetic foot ulcer	+	na
Prateek/2015	*odoratus*	1	Pericardial effusion	CKD on haemodialysis	−	Pan-drug resistant
Ali/2015	* Myroides* spp.	1	Canaliculitis	None	+	R: beta lactams and monobactams
Lahmer/2016	*odoratus*	1	Necrotizing pancreatitis, septic shock, multiorgan failure	Chronic alcoholic, possibly cirrhotic	+	S: cotrimoxazole and ciprofloxacin
Willems/2016	*odoratimimus*	1	Erysipelas and sepsis	Dog scratch,	−	S: levofloxacin, clindamycin, meropenem, tigecycline
Beharrysingh/2017	* Myroides* spp.	1	Bacteraemia and cellulitis	Diabetes, H/O bilateral toe amputations, indwelling left chest wall catheter, unsanitary water exposure	−	S: meropenem, cotrimoxazole
Pompilio/2017	*odoratimimus*	1	Recurrent calcaneal ulcer	Trauma, diabetes, H/O skin graft at same site	−	S: imipenem
Licker/2018	*odoratimimus*	4	UTI	1 Post-renal transplant on immunosuppression with urinary catheter; 1 DKA with urinary catheter; 1 COPD on long-term steroids, H/O TURP with urinary catheter; 1 post-cancer-surgery with ureterostomy tube	3−, 1+	S: minocycline
Ahamed/2018	*odoratimimus*	1	UTI	ESRD on haemodialysis	+	Pan-drug resistant
Lorenzin/2018	*odoratimimus*	1	Recurrent macroscopic haematuria	ESRD on haemodialysis, diabetes, indwelling urinary catheter	−	S: cotrimoxazole
LaVergne/2019	*injenensis*	1	Cellulitis	Alcoholic cirrhosis	+	S: ampicillin/sulbactam, piperacillin/tazobactam, ciprofloxacin, levofloxacin, meropenem
Meyer/2019	* Myroides* spp.	1	Cellulitis and bacteraemia	Diabetes mellitus, COPD on long-term steroids, trauma, animal exposure to wounds	−	S: ciprofloxacin, levofloxacin, piperacillin/tazobactam, imipenem
Mohapatra/2019	* Myroides* spp.	1	Empyema due to ruptured liver abscess	Chronic alcoholic, possibly cirrhotic	−	S: amoxiclav, piperacillin/tazobactam, carbapenems and ciprofloxacin
Lu/2020	*odoratimimus*	1	Catheter related bloodstream infection	Indwelling catheter,	+	S: cefoperazone/sulbactam
Foo/2020	* Myroides* spp.	1	Septic shock	Diabetes, foot ulcer, exposure to soil	−	S: meropenem
Perez-Lazo/2020	*phaeus*	1	Bacteraemia	CKD on haemodialysis, multiple myeloma on chemotherapy, indwelling catheter	−	S: Piperacillin/tazobactam
Kutlu/2020	*odoratimimus*	6	3 UTI, 3 bladder colonization	6 indwelling urinary catheter and diabetes; 1 COPD on long-term steroids, 1 ESRD	−	Pan-drug resistant
Yang/2020	*odoratimimus*	22	4 Bacteraemia; 18 incidental	22 prolonged hospitalization, post-operative, urinary catheterization	−	S: fluoroquinolones
Vempuluru/2021	* Myroides* spp.	1	Canaliculitis	na	+	S: chloramphenicol
Mahendran/2021	* Myroides* spp.	1	UTI	DKA	−	S: minocycline
Beathard/2021	* Myroides* spp.	1	Cellulitis and bacteraemia	CKD, diabetes	−	S: ciprofloxacin, meropenem
O'Neal/2022	*odoratus*	2	1 VAP, 1 bacteraemia	1 prolonged hospitalization, invasive procedures, indwelling catheters; 1 postoperative, prolonged hospitalization, indwelling catheters	+	S: minocycline, ceftazidime/avibactam
Faraz/2022	* Myroides* spp.	1	Recurrent UTI	Diabetes, post-renal transplant, repeated self-catheterization due to neurogenic bladder	−	Pan-drug resistant
Kurt/2022	*odoratimimus*	1	Bacteraemia	Indwelling catheters, Covid 19 pneumonia	−	Pan-drug resistant

AST, antimicrobial susceptibility testing; CKD, chronic kidney disease; HBV, hepatitis B virus; HCV, hepatitis C virus; UTI, urinary tract infection; na, not applicable; D-J stent, double J stent; H/O, history of; DKA, diabetic ketoacidosis; COPD, chronic obstructive pulmonary disease; TURP, transurethral resection of prostate; ESRD, end-stage renal disease; VAP, ventilator associated pneumonia.

## Case presentation

All urine samples received in the department of microbiology having significant bacteriuria for *Myroides* spp. were included in the study. For all cases, identification of the organism was done via the VITEK 2C system (bioMérieux). Antibiotic susceptibility testing was done using both manual (Kirby Bauer for disc diffusion and E-test for MICs) and automated (VITEK 2C) methods, following Clinical and Laboratory Standards Institute (CLSI) guidelines. Each isolate was sent to a reference laboratory for species identification using the matrix-assisted laser desorption ionization – time of flight (MALDI-TOF) Biotyper Sirius system (Bruker Daltonics). Sample size calculation using a power analysis was not done since this is a prospective observational study.

### Case 1

A 48-year-old male presented to the emergency department in an unconscious and intubated condition with a suspected brain injury. He was a known case of hypertension with type 2 diabetes mellitus and was on regular medications for the last 2 years. Magnetic resonance imaging of the brain revealed intracranial bleeding, following which he was immediately taken in for craniotomy. Requisite pre-operative preparation, including urinary catheter placement, was carried out. On the fourth day of the surgery, the patient experienced bouts of fever and increased white blood cell (WBC) counts. A urine culture was sent to the microbiology laboratory before empirical therapy was begun with intravenous ceftriaxone. Cultures reported *Myroides* spp. sensitive only to minocycline, and species identification yielded *M. odoratimimus* as the causative organism. Administration of minocycline resulted in an improvement in WBC counts, and urine cultures sent on the seventh day of surgery returned negative. This was our index case of *Myroides* spp. The patient’s condition worsened and eventually proved fatal, although not attributed to *Myroides* infection.

### Case 2

A 29-year-old female who was a case of post-partum haemorrhage (PPH) and gestational diabetes was brought into our centre in an intubated condition. The patient developed abdominal pain with oliguria and altered sensorium 4 days after her normal vaginal delivery, following which she was transferred to our hospital and catheterized on admission. Based on abnormal baseline laboratory parameters on arrival, the patient was immediately moved to the intensive care unit (ICU). An emergent abdominal computed tomography (CT) scan revealed abdominal wall cellulitis with cystitis, and empiric therapy with intravensour piperacillin-tazobactam was begun for the same. Cultures on day 0 and 6 revealed no growth. On day 9 of her hospital stay, a fever spike was noted for which investigations were requested, with urine microscopy revealing pyuria. A urine culture was sent to the microbiology laboratory for further evaluation, revealing extensively drug-resistant *Myroides* spp. growth with sensitivity to only minocycline. The antibiotic minocycline was added to ongoing treatment for 7 days and counts resolved to within normal limits.

### Case 3

A 41-year-old haemodynamically unstable diabetic male patient was referred to our hospital after having undergone multiple procedures including placement of a double-J (D-J) stent for a previous ureteric stricture and an emergent tracheal tube insertion. He presented with right-sided pleural effusion and was a known case of tuberculosis receiving modified anti-tubercular therapy, due to altered liver and kidney function. On admission, initial laboratory investigations revealed raised procalcitonin values, and a provisional diagnosis of sepsis and tubercular empyema was made. He was then moved to the ICU and catheterized for monitoring of kidney function. The patient had a fever spike on the eighth day, with urine microscopy revealing pyuria. A urine culture was sent to the laboratory and extensively drug-resistant *Myroides* spp. were isolated which were only sensitive to minocycline. Urine infection subsided by day 7 of minocycline administration and repeat cultures showed no further growth after cessation of treatment.

### Case 4

A 55-year-old male was referred to our centre, having been admitted for breathlessness of sudden onset for 3 days. He arrived in an intubated and unconscious condition and was catheterized on ICU admission, revealing an oliguric state. He was a known case of type 2 diabetes mellitus for 7 years and hypertension for 2 years treated with insulin and antihypertensive medications. On initial workup and examination, a diagnosis of acute chronic kidney disease, lower respiratory tract infection, cardiogenic shock and multiorgan failure was made. Emergent treatment was begun. Ultrasonography of the abdomen revealed right-sided pleural effusion and bilaterally raised renal echogenicity. Initial blood and urine culture reports yielded no growth. On day 8 of admission, a fever spike was noted with raised WBC counts and pus cells on urine microscopy. Urine cultures were sent to guide empiric therapy and *Myroides* spp. were reported with susceptibility only to minocycline. The treatment plan was updated, and minocycline was administered for 7 days. Repeat cultures reported no growth and counts returned to normal. However, the condition of the patient deteriorated, and he died due to complications not attributable to the infection.

### Case 5

A 69-year-old male was admitted to the emergency department with oliguria for the past 20 days and recent onset of disorientation and appetite loss over the previous 24 h. He was a known case of hypertension, type 2 diabetes, and chronic kidney disease for the last 5 years with a D-J stent in place. The patient was primarily managed in the emergency department where he was intubated, catheterized and then moved to the ICU. After further evaluation, a provisional diagnosis of acute liver failure and acute kidney injury with obstructive uropathy was made. Within 2 days of catheterization, the patient had a fever spike and raised WBC counts. Samples were sent for urine culture, which reported multi-drug resistant *Myroides* spp. Susceptibility was noted only to minocycline, similar to all our other cases. After administration of minocycline for 7 days, repeat cultures revealed no growth.

## Literature review

We conducted a literature search on PubMed using a mix of controlled terms and free text searching, with the string ‘((Flavobacteriaceae Infections) AND Myroides) OR (Myroides AND Infections)’ which yielded 72 results. Papers of English language that presented clinical Myroides spp. infection were considered for inclusion. Forty-two papers describing a total of 97 cases were included (Table 1).

## Discussion

Our study is the latest in a series of recent studies that have found *Myroides* spp. to be almost pan-drug resistant [13, 15, 24, 25, 28, 30, 35–37]. This is the first case from Western India, and the third case worldwide that shows *Myroides* sensitivity only to minocycline [24, 35]. Analysis of the trends behind the occurrence of *Myroides* infection revealed a wide swathe of possible causes, but a trend emerged with infections occurring in hospitalized patients. Our case series (Table 2) is similar to other cases [16, 19] in that there was a cluster of healthcare-associated urinary tract infections in patients with multiple comorbidities. All five cases had an indwelling device and were diabetic, pointing to these two being possible contributory factors to infection. Further investigation of contributory factors in the pre-existing literature revealed similar aetiologies in numerous cases. We elucidate this in Table 3, a clinically useful tool that portrays, at a glance, the risk factors for *Myroides* infection and drug classes most likely to be effective in treatment. This can help guide clinicians to suspect *Myroides* infection and initiate appropriate antibiotic therapy while keeping in mind local sensitivity patterns.

**Table 2.** Summary of case series

Case no./age (Years)/sex	Location of admission	Collection date (of positive sample)	Possible contributory factors	Immune status	Presence of indwelling device	Infection	Treatment	Outcome
C1/48/M	SICU	Day 4	Diabetes, invasive procedure	–	Urinary catheter, endotracheal tube	UTI	Minocycline	Fatal (unrelated to infection)
C2/29/F	SICU	Day 9	Gestational diabetes, prolonged hospitalization	–	Urinary catheter, endotracheal tube	UTI	Minocycline	Cured
C3/41/M	CCMICU	Day 8	Diabetes, CKD, tubercular empyema, prolonged hospitalization, invasive procedure	–	D-J stent, tracheal tube, urinary catheter, intracranial drain	UTI	Minocycline	Cured
C4/55/M	CCMICU	Day 8	Diabetes, CKD, multiorgan failure, prolonged hospitalization	–	Urinary catheter, endotracheal tube, central line	UTI	Minocycline	Fatal (unrelated to infection)
C5/69/M	SICU	Day 2	Diabetes, CKD, hydronephrosis with ureteric stricture, sepsis, prolonged hospitalization	–	D-J stent, tracheal tube, urinary catheter	UTI	Minocycline	Cured

CKD, chronic kidney disease; SICU, surgical intensive care unit CCMICU, critical care medicine intensive care unit; UTI, urinary tract infection; D-J stend, double J stent.

**Table 3.** Clinical support tool

Risk factors for infection	Targeted therapeutic options*
Indwelling catheters/devices Prolonged hospitalization Invasive/surgical procedures Diabetes CKD	Chloramphenicol Levofloxacin Cotrimoxazole Meropenem Minocycline

*Sensitive in >50 % of cases in the existing literature.

CKD, chronic kidney disease.

*Myroides* has classically been considered a ubiquitous organism and an opportunistic pathogen associated with infection only in immunocompromised patients (Table 1) or those having multiple comorbidities [34]. Cases due to animal exposure have been reported [20, 22] and unsanitary environmental exposure via water or soil has been suspected as a contaminant [7, 9, 18, 21, 34, 38–40]. Yet, attempts at identifying the source of this infection in nosocomial settings have been largely unsuccessful [16, 19]. Of note is that infections in immunocompetent individuals are increasingly appearing in the literature [8–10, 14–16, 18–21, 24, 25, 27, 31, 32, 41, 42]. This throws into question the clinical presumption that *Myroides* infection is always opportunistic, and due diligence needs to be given to this emerging extensively drug-resistant pathogen.

Initiating appropriate treatment for *Myroides* infection is challenging due to resistance to commonly used antimicrobial agents. *Myroides* can auto-aggregate and co-aggregate to form biofilms and have strong adherence and hydrophobicity [23, 26]. These properties along with chromosomally encoded beta-lactamases are hypothesized to confer drug resistance to most *Myroides* species [43]. More research is needed to elucidate the exact mechanisms of resistance in this organism [44, 45]. Antibiotic susceptibility testing on our isolates using the VITEK 2C system revealed resistance to penicillins, cephalosporins, norfloxacin, ciprofloxacin, aztreonam, amikacin, gentamicin, imipenem, meropenem, colistin and polymyxin. Susceptibility only to minocycline was seen.

The emergence of such extensive drug resistance led us to analyse the existing literature for trends in risk factors and antimicrobial sensitivity, which resulted in the development of the clinical tool presented (Table 3). All papers retrieved through the literature search were used to mine data, and this gave startling results. The five most likely risk factors – indwelling catheters/devices, prolonged hospitalization, invasive/surgical procedures, diabetes and chronic kidney disease – were present in 56, 36, 35, 21 and 12 % of cases respectively. This finding underlines the high chance of infection that these risk factors confer on patients. Targeted therapeutic options were selected if they were sensitive in at least 50 % of the studies in which they were reported. Antibiotics that were not reported in at least three papers were not included, and intermediate sensitivity was not factored into the analysis. This led us to elucidate the top five therapeutic options that are useful in *Myroides* infections. Minocycline, though having the highest sensitivity percentage of 100 %, was placed at the end of the table since it was reported in just four studies, in contrast to the average of 10 studies for other antibiotic choices. More case reports which include minocycline in their antimicrobial susceptibility testing are required before it can be recognized as the drug of choice. Spanik *et al*. [8] found that source removal via changing catheters led to the resolution of *Myroides* infection without specific antimicrobial therapy. This therapeutic modality can be explored in subsequent studies. Timely and appropriate identification of *Myroides *, through antimicrobial testing and antibiotic susceptibility testing to better guide therapeutic decisions and interventions, is imperative, given their potential to cause outbreaks, non-healing of wounds and prolonged duration of hospital stay.

There are a few limitations of the present study. While conducting the literature review, we restricted our search to only MEDLINE. Expanding the scope of the search will yield additional cases, yet were beyond the scope of this study. A significant number of cases retrieved were of urinary tract infections, including our own, which could explain the high prevalence of catheterization and its subsequent association with *Myroides* infection. Future research in this area should aim to systematically review all available reports to glean information existing in different databases.

## References

[1] Hugo CJ, Bruun B, Jooste PJ, Dworkin M, Falkow S, Rosenberg E, Schleifer K-H, Stackebrandt E (2006). The Prokaryotes.

[2] Holmes B, Snell JJ, Lapage SP (1979). *Flavobacterium odoratum*: a species resistant to a wide range of antimicrobial agents. J Clin Pathol.

[3] Davis JM, Peel MM, Gillians JA (1979). Colonization of an amputation site by *Flavobacterium odoratum* after gentamicin therapy. Med J Aust.

[4] Macfarlane DE, Baum-Thureen P, Crandon I (1985). *Flavobacterium odoratum* ventriculitis treated with intraventricular cefotaxime. J Infect.

[5] Hsueh PR, Wu JJ, Hsiue TR, Hsieh WC (1995). Bacteremic necrotizing fasciitis due to *Flavobacterium odoratum*. Clin Infect Dis.

[6] Ferrer C, Jakob E, Pastorino G, Juncos LI (1995). Right-sided bacterial endocarditis due to *Flavobacterium odoratum* in a patient on chronic hemodialysis. Am J Nephrol.

[7] Bachman KH, Sewell DL, Strausbaugh LJ (1996). Recurrent cellulitis and bacteremia caused by *Flavobacterium odoratum*. Clin Infect Dis.

[8] Spanik S, Trupl J, Krcmery V (1998). Nosocomial catheter-associated *Flavobacterium odoratum* bacteraemia in cancer patients. J Med Microbiol.

[9] Green BT, Green K, Nolan PE (2001). *Myroides odoratus* cellulitis and bacteremia: case report and review. Scand J Infect Dis.

[10] Motwani B, Krezolek D, Symeonides S, Khayr W (2004). *Myroides odoratum* cellulitis and bacteremia. Infectious Diseases in Clinical Practice.

[11] Deepa R, Venkatesh KG, Parveen JD, Banu ST, Jayalakshmi G (2014). *Myroides odoratus* and *Chryseobacterium indologenes*: two rare isolates in the immunocompromised. Indian J Med Microbiol.

[12] Crum-Cianflone NF, Matson RW, Ballon-Landa G (2014). Fatal case of necrotizing fasciitis due to *Myroides odoratus*. Infection.

[13] Prateek S, Gupta P, Mittal G, Singh AK (2015). Fatal case of pericardial effusion due to *Myroides odoratus*: a rare case report. J Clin Diagn Res.

[14] Lahmer T, Beitz A, Ehmer U, Schmid RM, Huber W (2016). Septic shock due to *Myroides odoratus* in a medical intensive care unit patient with severe necrotising pancreatitis. Anaesth Intensive Care.

[15] O’Neal M, Labay CE, Harris JE, Musick WL, Cernoch PL (2022). Extensively drug-resistant *Myroides odoratus* in critically Ill patients: a case series and literature review. Case Reports in Infectious Diseases.

[16] Yağci A, Cerikçioğlu N, Kaufmann ME, Malnick H, Söyletir G (2000). Molecular typing of *Myroides odoratimimus* (*Flavobacterium odoratum*) urinary tract infections in a Turkish hospital. Eur J Clin Microbiol Infect Dis.

[17] Bachmeyer C, Entressengle H, Khosrotehrani K, Goldman G, Delisle F (2008). Cellulitis due to *Myroides odoratimimus* in a patient with alcoholic cirrhosis. Clin Exp Dermatol.

[18] Benedetti P, Rassu M, Pavan G, Sefton A, Pellizzer G (2011). Septic shock, pneumonia, and soft tissue infection due to *Myroides odoratimimus*: report of a case and review of Myroides infections. Infection.

[19] Ktari S, Mnif B, Koubaa M, Mahjoubi F, Ben Jemaa M (2012). Nosocomial outbreak of *Myroides odoratimimus* urinary tract infection in a tunisian hospital. J Hosp Infect.

[20] Maraki S, Sarchianaki E, Barbagadakis S (2012). *Myroides odoratimimus* soft tissue infection in an immunocompetent child following a pig bite: case report and literature review. Braz J Infect Dis.

[21] Endicott-Yazdani TR, Dhiman N, Benavides R, Spak CW (2015). *Myroides odoratimimus* bacteremia in a diabetic patient. Proc.

[22] Willems P, Muller J, Verhaegen J, Saegeman V, Desmet S (2017). How to treat a fulminant erysipelas and sepsis caused by *Myroides odoratimimus*: case report and literature review. Acta Clin Belg.

[23] Pompilio A, Galardi G, Gherardi G, Verginelli F, Geminiani C (2018). Infection of recurrent calcaneal ulcer caused by a biofilm-producer *Myroides odoratimimus* strain. Folia Microbiol.

[24] Licker M, Sorescu T, Rus M, Cirlea N, Horhat F (2018). Extensively drug-resistant *Myroides odoratimimus* - a case series of urinary tract infections in immunocompromised patients. Infect Drug Resist.

[25] Ahamed I, Annapandian VM, Muralidhara KD (2018). *Myroides odoratimimus* urinary tract infection. Saudi J Kidney Dis Transpl.

[26] Lorenzin G, Piccinelli G, Carlassara L, Scolari F, Caccuri F (2018). *Myroides odoratimimus* urinary tract infection in an immunocompromised patient: an emerging multidrug-resistant micro-organism. Antimicrob Resist Infect Control.

[27] Lu Y, Xia W, Zhang X, Ni F, Mei Y (2020). A confirmed catheter-related blood stream infection (CRBSI) in an immunocompetent patient due to *Myroides odoratimimus*. Case Report and Literature Review Infect Drug Resist.

[28] Kutlu HH, Avcı M, Dal T, Arı O, Durmaz R (2020). A healthcare-associated outbreak of urinary tract infections due to *Myroides odoratimimus*. Jpn J Infect Dis.

[29] Yang S, Liu Q, Shen Z, Wang H, He L (2020). Molecular epidemiology of *Myroides odoratimimus* in nosocomial catheter-related infection at a general hospital in China. Infect Drug Resist.

[30] Kurt AF, Mete B, Houssein FM, Tok Y, Kuskucu MA (2022). A pan-resistant *Myroides odoratimimus* catheter-related bacteremia in a COVID-19 patient and review of the literature. Acta Microbiol Immunol Hung.

[31] Kim DS, Paek J, Shin JH, Kim DW, Jung MY (2012). Genome sequence of *Myroides injenensis* M09-0166(T), isolated from clinical specimens. J Bacteriol.

[32] LaVergne S, Gaufin T, Richman D (2019). *Myroides injenensis* bacteremia and severe cellulitis. Open Forum Infect Dis.

[33] Pérez-Lazo G, Morales-Moreno A, Soto-Febres F, Jove-Químper H, Morales-Castillo L (2020). First report of *Myroides phaeus* bacteraemia identified by Polymerase chain reaction and genetic sequencing. IDCases.

[34] Beharrysingh R (2017). *Myroides* bacteremia: a case report and concise review. IDCases.

[35] Mahendran AJ, Agrawal S, Rastogi N, Gupta N (2021). *Myroides*: a rare but hard-to-crack villain in a critical care setup. Indian J Crit Care Med.

[36] Beathard WA, Pickering A, Jacobs M (2021). *Myroides* cellulitis and bacteremia: a case report. IDCases.

[37] Faraz A, Fathima K, Kazmi SY, Al Motery AS, Ghaffar UB (2022). Recurrent urinary tract infection in a renal transplant patient by pan-resistant *Myroides* spp. J Coll Physicians Surg Pak.

[38] Wang Q, Wang P, Yang Q (2018). Occurrence and diversity of antibiotic resistance in untreated hospital wastewater. Sci Total Environ.

[39] Douce RW, Zurita J, Sanchez O, Cardenas Aldaz P (2008). Investigation of an outbreak of central venous catheter-associated bloodstream infection due to contaminated water. Infect Control Hosp Epidemiol.

[40] Thomas M, Padmini SB, Govindan VK, Appalaraju B (2007). Oerskovia turbata and *Myroides* species: rare isolates from a case of acalculus cholecystitis. Indian J Med Microbiol.

[41] Ali MJ, Joseph J, Sharma S, Naik MN (2017). Canaliculitis with isolation of *Myroides* species. Ophthalmic Plast Reconstr Surg.

[42] Vempuluru VS, Mitra S, Tripathy D, Mohapatra S, Rath S (2021). Isolation of unusual bacteria in canaliculitis: a series of four cases. Saudi J Ophthalmol.

[43] Gunzer F, Rudolph WW, Bunk B, Schober I, Peters S (2018). Whole-genome sequencing of a large collection of *Myroides odoratimimus* and *Myroides odoratus* isolates and antimicrobial susceptibility studies. Emerg Microbes Infect.

[44] Xu S, Chen Y, Fu Z, Li Y, Shi G (2018). New subclass B1 metallo-β-lactamase gene from a clinical pathogenic *Myroides odoratus* strain. Microb Drug Resist.

[45] Hu S, Yuan S, Qu H, Jiang T, Zhou Y (2016). Antibiotic resistance mechanisms of *Myroides* sp. J Zhejiang Univ Sci B.

